# The Equilibrium, Kinetics, and Thermodynamics Studies of the Sorption of Methylene Blue from Aqueous Solution Using Pulverized Raw Macadamia Nut Shells

**DOI:** 10.1155/2020/8840666

**Published:** 2020-05-30

**Authors:** Joshua N. Edokpayi, Samson O. Alayande, Ahmed Adetoro, John O. Odiyo

**Affiliations:** ^1^Hydrology and Water Resource Department, University of Venda, Thohoyandou, South Africa; ^2^Department of Physical Sciences, First Technical University, Ibadan, Nigeria; ^3^Department of Chemical Sciences, Yaba College of Technology, Lagos, Nigeria

## Abstract

In this study, the potential for pulverized raw macadamia nut shell (MNS) for the sequestration of methylene blue from aqueous media was assessed. The sorbent was characterized using scanning electron microscopy for surface morphology, functional group analysis was performed with a Fourier-transform infrared spectrometer (FT-IR), and Brunauer–Emmett–Teller (BET) isotherm was used for surface area elucidation. The effects of contact time, sorbent dosage, particle size, pH, and change in a solution matrix were studied. Equilibrium data were fitted using Temkin, Langmuir, and Freundlich adsorption isotherm models. The sorption kinetics was studied using the Lagergren pseudo-first-order, pseudo-second-order, Elovich, and intraparticle diffusion models. The feasibility of the study was established from the thermodynamic studies. A surface area of 2.763 m^2^/g was obtained. The equilibrium and kinetics of sorption was best described by the Langmuir and the pseudo-second-order models, respectively. The sorption process was spontaneous (−Δ*G*^0^=28.72 − 31.77 kJ/mol) and endothermic in nature (Δ*H*^0^=17.45 kJ/mol). The positive value of ΔS^0^ (0.15 kJ/molK) implies increased randomness of the sorbate molecules at the surface of the sorbent. This study presents sustainable management of wastewater using MNS as a potential low-cost sorbent for dye decontamination from aqueous solution.

## 1. Introduction

Freshwater resources are increasingly under threat from pollution due to anthropogenic activities. Although freshwater is unevenly distributed across the globe, it remains a major resource for livelihood and economic growth. The threat to freshwater quality is majorly caused by human activities and lifestyles. The discharge of untreated and partially treated wastewater remains a major point source of surface water pollution [1]. The impact of polluting freshwater includes loss of source of livelihood, undue stress to aquatic organisms, and negative impacts on the environment [2, 3].

One of the recalcitrant pollutants to surface water bodies is dyes. The release of small quantity of dyes in wastewater has a far-reaching effect on the aesthetic value of the receiving watershed. Most dyes are synthetically produced and, therefore, not biodegradable [4, 5]. Apart from being persistent in the environment, they are also hazardous, causing physiological changes or death to benthic organisms including fish. They prevent sunlight from penetrating a water body, thereby affecting photosynthetic plants in the aquatic ecosystem [6, 7]. Their presence usually leads to a decrease in dissolved oxygen in the aquatic ecosystem. Dyes production are on the increase due to their application in various industrial processes and products which include but are not limited to textile, cosmetics, tanning, pharmaceutical, pulp, and paper industries. Many textile and allied industries unfortunately do not treat their effluents to regulatory standards before releasing the dye-rich effluents into the receiving watersheds [3, 8]. Dyes are recalcitrant in nature and very difficult to remove due to their high solubility and low biodegradability.

Although several conventional methods exist for their removal such as coagulation and floatation, ozonation, membrane separation, and commercially available activated carbon, they often suffer setbacks due to their inability to remove certain dyes from the water stream [9–11]. Also, the cost of installation, energy consumption and expertise often limit their use. There is a continuous search for low-cost, eco-friendly, and sustainable materials for dye pollution control. Attention has shifted to the use of agricultural waste materials for the removal of dyes from environmental media. Several plant-based materials have been used which showed promising results; however, the continuous availability of those materials has limited their potential for use [5, 12, 13].

South Africa is the third largest producer of macadamia in the world. Most of the fruits and oils are often sold in the international market [14]. Macadamia is widely used in the snacks and food industries for baking, making ice cream, and as confectionery. It is also widely used in the cosmetic industry. One of the waste materials for macadamia processing is the nut shells. The nut shells have been used as a source of energy for local bricks industries in South Africa. It has also been used to augment some building materials and as source of fertilizers.

Few authors have reported on the methods of making activated carbon from macadamia nut shell (MNS) [15–17]. Also, few reports have investigated the use of activated carbon from MNS for hexachromium removal [16], tetracycline removal [18] phenol removal [19], and reactive dyes [20]. No study, however, has reported the use of the raw natural product for the decontamination of methylene blue. Thus, this study investigated the impact of change in water chemistry on the sorption of methylene blue onto raw pulverized MNS. This study is, therefore, aimed at evaluating the potential of South African macadamia nut shell for decontamination of hazardous dye from aqueous solution; the force needed to break the nut shell is also reported.

## 2. Materials and Method

### 2.1. Adsorbate Preparation

Methylene blue (MB) was purchased from Fisher Scientific from the United States of America. 1000 mg/L stock solution was prepared following standard methods. Working solutions were made by the dilution method from the prepared stock solution. 0.1 M NaOH and HCl were used for pH adjustment.

### 2.2. Sorbent Preparation

**Figure 1:**
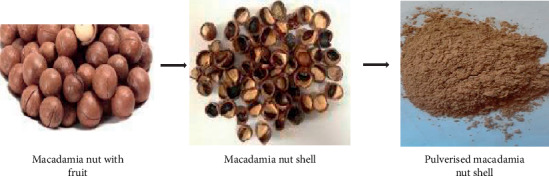
Stages of adsorbent preparation.

Waste macadamia shells were collected from a processing farm in Vhembe District, Limpopo Province of South Africa. The hard shells were separated from the valuable nuts which are very rich in essential oils. The shells were washed with deionised water and oven dried (Eco Therm, Labotec) for 24 hrs at 105°C (Figure 1). The dried shells were pulverized to fine particles using a pulveriser (Retsch RS 200). Mechanical sieves (King-Test VB 200/300) were used to obtain various fractions of the adsorbent.

### 2.3. Instrumentation

MB in aqueous solution was analysed using a UV spectrophotometer (Orion Aquamate 7000, Thermoscientific) at a predetermined wavelength of 664 nm. The mechanical property of the nut was determined using Computerized Ingstrom Machine. Fourier transform infrared (FT-IR) spectra of the sorbent were obtained using a Perkin Elmer 100 FT-IR spectrophotometer (Waltham, MA, USA) with accessories. The spectra were scanned over the wave number range of 4500 to 400 cm^−1^. The surface area and pore width were determined by the N_2_ gas Brunauer–Emmett–Teller method of analysis using a Micromeritics Chemisorption ASAP 2020 analyzer (Norcross, GA, USA). A temperature-controlled shaking water bath (EcoBath, Labotec) was used to agitate the solutions at specific time and temperature. An Orion pH meter (Thermo Orion VersaStar) was used to measure the pH of the solution. The surface morphology of the sorbent was analysed using scanning electron microscopy (SEM). The sample was crushed, mounted on a stud, carbon coated, and irradiated with a beam of electrons at 20 Kv, and the resulting micrograph was recorded.

### 2.4. Batch Sorption Studies

Several sorption experimental drivers were investigated to determine their effect of the sorption process.

The effects of sorption time were investigated by varying the sorption time between 5 and 270 minutes using three different dosages (1.25, 2.5, and 3.75 g/L) of MNS with an initial MB concentration of 30 mg/L. The samples were placed in a shaking water bath set at 303 K and 250 rpm. Samples were withdrawn from the shaking water bath at predetermined intervals, centrifuged at 250 rpm and analysed for residual MB concentration at 664 nm.

Similarly, the effect of sorbent dosage was performed using 0.25–3.75 g/L of MNS with two initial MB concentrations (30 and 70 mg/L). The solutions were equilibrated for 3 hours at 250 rpm. The experiment was performed at 303, 313, and 323 K. All the samples were then centrifuged and subsequently analysed using a UV-Vis spectrophotometer. A control sample without the sorbent was also ran for quality control.

The percentage of MB removed was calculated using the relation in equation (1), and the quantity of MB sorbed was calculated using equation (2):

(1) $$\begin{array}{c} \text{removal }\left(\%\right)=\frac{C_{\text{e}}-C_{0}}{C_{\text{o}}}\times 100, \end{array}$$

(2) $$\begin{array}{c} {\text{q}}_{\text{e}}=\frac{\text{V}\left(C_{O\;}-C_{e}\right)}{M}, \end{array}$$

where q_e_ is the amount of adsorbate adsorbed at equilibrium (mg/g), *C*_o_ and *C*_e_ were the initial and the equilibrium concentrations in mg/L, respectively, V is the volume in liters of the solution used during the experiment, and *M* is the mass of the adsorbent in Gram.

The effects of solution pH were elucidated by varying the pH of the dye solution (30 mg/L) between 2 and 11 using three different dosages (0.25, 0.5, and 1.25 g/L). The other experimental procedure was the same as stated for the effects of dosage. The sorbent particle size was varied between 10–124 *µ*m (referred to as <125 *µ*m) and 125–250 *µ*m (referred to as >125 *µ*m), to investigate their impact of the sorption process. The final MB concentration was analysed after sorption.

The influence of change in water chemistry was investigated by preparing MB solution with water collected from the Mutale River in Vhembe District, South Africa. The water chemistry of the river water was analysed for basic physicochemical parameters using standard methods. The result obtained was compared to that of deionised water.

### 2.5. Isotherm Study of the Sorption Processes

The data obtained from the batch sorption studies were fitted into three equilibrium isotherms. The Langmuir, Freundlich, and Temkin adsorption isotherms were used.

The Langmuir isotherm often estimates the maximum adsorption capacity corresponding to complete monolayer coverage on the adsorbent surface. The linearized equation is represented as follows [21]:

(3) $$\begin{array}{c} \frac{1}{{\text{q}}_{\text{e}}}=\frac{1}{{\text{q}}_{\text{max}}}+\left(\frac{1}{b{\text{q}}_{\text{max}}}\right)\frac{1}{{\text{C}}_{\text{e}}}, \end{array}$$

where *C*_e_ is the equilibrium concentration of MB (mg/L), q_e_ is the quantity of MB dye adsorbed at equilibrium (mg/g), q_max_ is the maximum amount of MB sorbed (mg/g), and *b* is the adsorption constant (L/mg). A plot of 1/*C*_e_ versus 1/q_e_ will give a straight line, and q_max_ and *b* can be obtained from the intercept and slope of the plot, respectively.

The Freundlich model assumes a heterogeneous sorption surface with unequal available sites with different energies of sorption. Its linearized equation is given as follows [22]:

(4) $$\begin{array}{c} {\text{log}}_{{\text{q}}_{\text{e}}}{=\text{log}}_{{\text{K}}_{\text{f}}}+\frac{1}{n_{\text{f}}}{\text{logc}}_{\text{e}}. \end{array}$$

The plot of log q_e_ versus log *C*_e_ provides the intercept *K*_f_ and the slope 1/*n*_f_, where q_e_ is the amount of adsorbate adsorbed at equilibrium (mg/g), *C*_e_ is the equilibrium concentration of adsorbate (mg/L), *K*_f_ is the Freundlich constant, and *n*_f_ is the Freundlich exponent.

The Temkin isotherm model assumes that the heat of adsorption of all the molecules on the adsorbent surface layer would decrease linearly with coverage due to adsorbate-adsorbate interactions, and this fall in the heat of adsorption is not logarithmic as implied in the Freundlich equation [23]. The linearized form of the Temkin isotherm is represented as

(5) $$\begin{array}{c} {\text{q}}_{\text{e}}{=\text{BlnK}}_{\text{T}}+\text{Bln}C_{\text{e}}, \end{array}$$

where q_e_ is the amount of adsorbate adsorbed at equilibrium (mg/g), B is the constant related to the heat capacity (L/mg), *R* is the universal gas constant (8.314 J/mol·K), T is the absolute temperature (K), K_T_ is the equilibrium binding constant (L/mg), and *C*_e_ is the equilibrium concentration of adsorbate (mg/L).

A plot of q_e_ against log *C*_e_ is linear, and the constant B (L/g) and *K*_T_ will be determined from the slope and intercept of the plots, respectively.

### 2.6. Thermodynamic Study of the Sorption Processes

In order to determine the thermodynamic feasibility of the sorption process, the standard Gibbs free energy change (ΔG^0^), the standard entropy change (ΔS^0^), and the standard enthalpy change (ΔH^0^)  were calculated. ΔG^0^ was determined from the relation below:

(6) $$\begin{array}{c} \left(\Delta {\text{G}}^{0}\right)=-{\text{RT In K}}_{0}, \end{array}$$

where K_0_ is the equilibrium constant (L/mol) determined from the Langmuir constant *b*. ΔS^0^ and ΔH^0^ were estimated using the van't Hoff equation [24]:

(7) $$\begin{array}{c} {\text{InK}}_{0}=\frac{\left(\Delta {\text{S}}^{0}\right)\;}{\text{R}}-\frac{\Delta {\text{H}}^{0}}{\text{RT}}, \end{array}$$

where T is the absolute temperature (K) and *R* is the gas constant (8.314 J·mol^−1^·K^−1^). The plot of InK_0_ as a function of 1/T should give a linear relationship with the slope of ΔH^0^/R and an intercept of ΔS^0^/R.

### 2.7. Kinetic Study of the Adsorption Processes

The kinetics of sorption describes the rate of MB uptake on MNS, and this rate controls the equilibrium time. The pseudo-first-order, pseudo-second-order, Elovich, and intraparticle diffusion models were used in this study. The linearized forms of these models are given in equations (8)–(11) [25–28].

The Lagergren pseudo-first-order kinetic model equation is given by

(8) $$\begin{array}{c} \text{Log }\left({\text{q}}_{\text{e}}-{\text{ q}}_{\text{t}}\right)={\text{Log q}}_{\text{e }}-\frac{{\text{k}}_{1}}{2.303}\text{t.} \end{array}$$

Pseudo-second-order kinetic equation is given by

(9) $$\begin{array}{c} \frac{\text{t}}{{\text{q}}_{\text{t}}}=\frac{1}{{\text{k}}_{2}{\text{q}}_{\text{e}}^{2}}+\frac{\text{t}}{{\text{q}}_{\text{e}}}. \end{array}$$

The Elovich model is given by

(10) $$\begin{array}{c} {\text{q}}_{t}=\frac{1}{\beta }\text{In}\left(\alpha \beta \right)+\;\frac{1}{\beta }\text{In}\left(t\right). \end{array}$$

The Webber Morris intraparticle model is given by

(11) $$\begin{array}{c} \text{q}=\text{Kp}.{\text{t}}^{1/2}, \end{array}$$

where q_e_ is quantity of MB sorbed at equilibrium, qt is the quantity of MB sorbed at time *t* in minutes, K_1_ is pseudo-first-order rate constant, K_2_ is pseudo-second-order rate constant, *a* is the initial adsorption rate (mg/gmin), *β* is the desorption constant (g/mg), and K_p_ (mg g^−1^ min^−1/2^) is the intraparticle diffusion rate constant.

## 3. Results and Discussion

**Figure 2:**
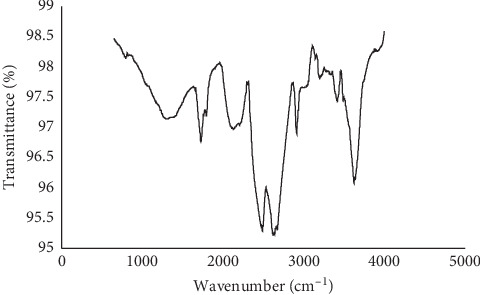
FT-IR spectra of MNS.

**Figure 3:**
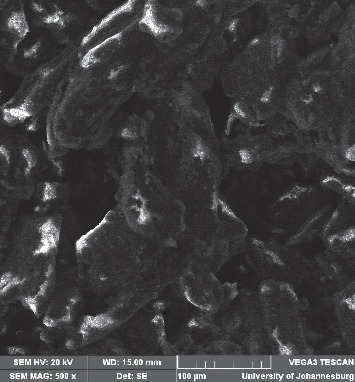
SEM micrograph of macadamia nut shell.

The FT-IR peaks recorded are presented in Figure 2. The hydroxyl, amine, and carbonyl groups were predominant in the sorbent (Table 1).  Figure 3 shows the SEM micrograph of the sorbent; surface particle agglomeration was observed with some slight voids capable of adsorbing the dye molecules.

**Figure 4:**
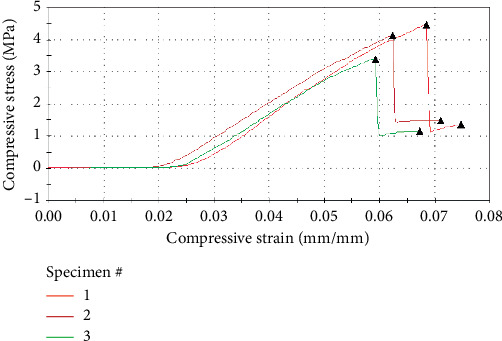
Mechanical property of macadamia nut.

The Brunauer–Emmett–Teller (BET) surface area of 2.763 m^2^/g (Table 2) was determined for the sorbent material. This is higher than the surface areas reported for *Dicerocaryum eriocarpum* leaves (1.85 m^2^/g), activated carbon made from macadamia nut shell (1.083 m^2^/g), and activated carbon from maize tassels (2.52 m^2^/g) [19, 29, 30], although other sorbent materials with higher surface area have been reported in the literature [18, 20]. The surface area value will favour strong affinity between a sorbate and sorbent. The sorbent is a macroporous material based on average pore diameter (Table 2). Due to this pore dimension, low energy is required for sorption of the sorbate by the sorbent [31, 32]. The load at break was 80.4 N while compressive stress at break was 0.15 MPa (Figure 4). Based on the stress-strain analysis of a nut under diametrical compression, material behaves in a brittle manner.

### 3.1. Batch Equilibrium

#### 3.1.1. Effects of Time

**Figure 5:**
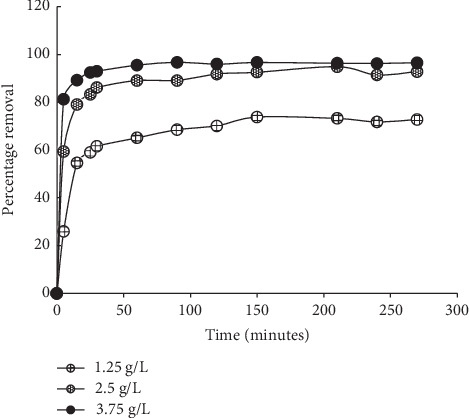
Effects of sorption time on MB uptake by MNS.

Sorption time usually plays a great role in the uptake of the sorbate by the sorbent. In this study, there was initially an increase in MB uptake as time progresses which can be linked to the available external surface on the sorbent. At 60 minutes of sorption time, >65% removal of initial MB concentration was recorded for a sorbent dose of 1.25 g/L (Figure 5). This increase continued until equilibrium was attained at 150 minutes (>95% uptake). After this point, no significant uptake of MB was recorded. This is due to the saturation of the sorbent surface. Similar results have been recorded in several other plant-based sorbents for MB sequestration [33, 34].

#### 3.1.2. Effects of Solution pH

**Figure 6:**
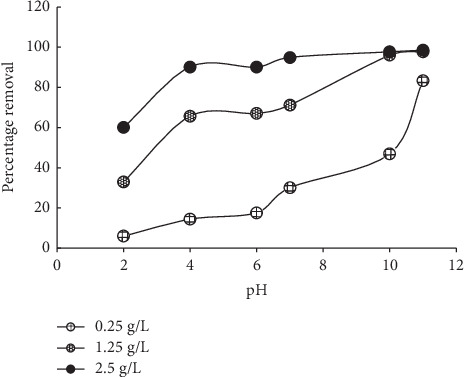
Effects of solution pH on the sorption of MB onto MNS.

Solution pH plays a great role in sorption reactions because it often determines the ionisation states of the functional groups present in a sorbent. In this study, higher sorption was recorded as the pH of the solution changes from acidic to more alkaline. The pH of highest sorption was 11 (Figure 6). Our previous study using Marula seed husks for uptake of MB showed high uptake at the pH of 6; however, this is slightly different with the findings of this study [6]. Similarly, Singh et al. [4] also showed that alkaline pH favored the sorption of MB onto *Ginkgo biloba* leaves, but 98% MB removal was recorded at the pH of 4 which negligibly increased up to the pH of 11.

#### 3.1.3. Effects of Sorbent Dosage

**Figure 7:**
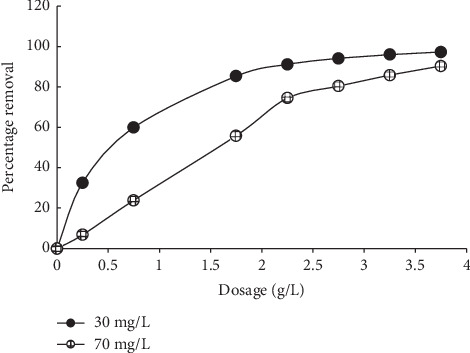
Effects of sorbent dosage on MB sorption onto MNS.

A general increase in MB uptake was recorded as the sorbent dosage increased from 0.5 to 4.0 g/L (Figure 7). This is due to the increase in the corresponding sorption sites present on the sorbent. Similar trends have been reported in the literature [17, 29, 35].

#### 3.1.4. Effects of Particle Size

**Figure 8:**
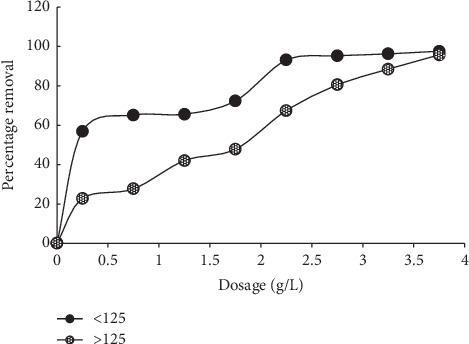
Effect of particle size on MB sorption onto MNS.

The smaller particle size (<125 um) of the sorbent recorded higher sorption than the bigger size of >125 um (Figure 8). This has been supported by several scholars that the smaller the particle size of the sorbent, the more the surface area. At higher sorbent dosage, the impact of particle size became negligible [6].

#### 3.1.5. Influence of Change in Water Chemistry

**Figure 9:**
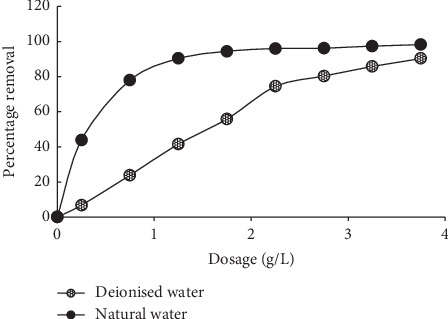
Influence of change in water chemistry on MB sorption on MNS.

The physicochemical water quality parameters of the Mutale River used in this study recorded the pH of 6.9, conductivity of 24.0 (*μ*S/cm), and turbidity of 14 NTU. The levels of major cations are sodium (4.66 mg/L), potassium (0.35 mg/L), calcium (2.15 mg/L), and magnesium (1.0 mg/L) while the levels of anions are sulphate (3.35 mg/L) and chloride (36.99 mg/L), respectively. The sorption was favored in natural water than in deionised water (Figure 9). This shows that the sorbent can be used on real wastewater or polluted water.

#### 3.1.6. Equilibrium Models

**Figure 10:**
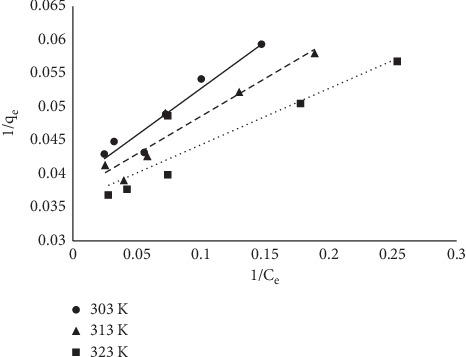
Langmuir plot for the sorption of MB onto MNS.

**Figure 11:**
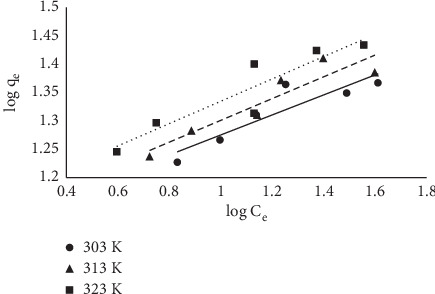
Freundlich plot for the sorption of MB onto MNS.

**Figure 12:**
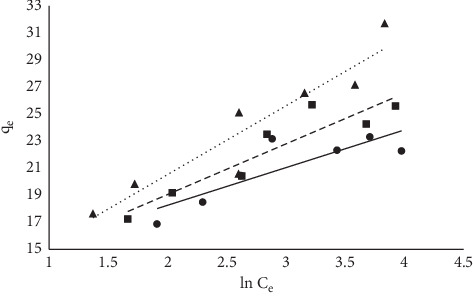
Temkin plot for the sorption of MB onto MNS.

The equilibrium data fitted into the three models are already described in equations (3)–(5). The linearized coefficient (*R*^2^) was used to determine the isotherm that best described the sorption process. Linear plots were obtained as described earlier, and *R*^2^ was determined from the plots (Figures 10–12). Maximum sorption capacity as well as the isotherm constant was derived from the slopes and intercepts of the respective plots (Table 3). In this study, the Langmuir equilibrium model best described the sorption process with an *R*^2^ factor of 0.93 at 303 K. The *R*^2^ factor for other models ranges from 0.73 to 0.88.

#### 3.1.7. Thermodynamics of the Sorption Process

**Figure 13:**
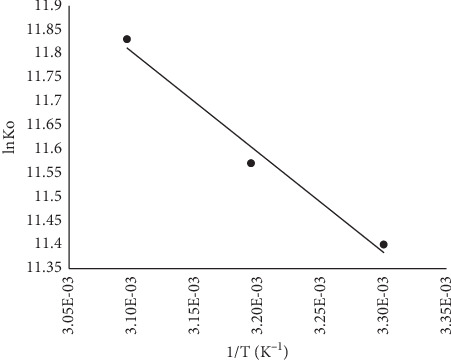
van't Hoff plot of the sorption of MB onto MNS.

This was estimated using the relation in equations (6) and (7), and negative values for ΔG were computed which imply that the sorption is spontaneous and thermodynamically feasible (Table 4). The values of ΔS and ΔH were obtained from the van't Hoff plot (Figure 13). The change in enthalpy was positive, which indicates an endothermic sorption process, while the change in entropy was also positive, implying an increased randomness of the sorbate molecules on the surface of the sorbent. Similar results have been reported by several scholars on MB sorption onto plant-based materials.

#### 3.1.8. Kinetic Models

**Figure 14:**
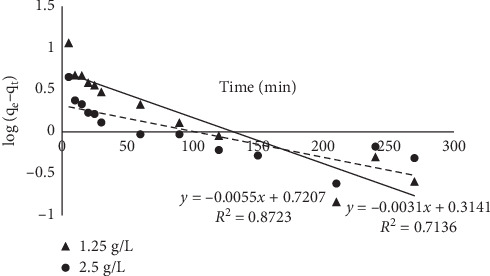
Pseudo-first-order plot for the sorption of MB onto MNS.

**Figure 15:**
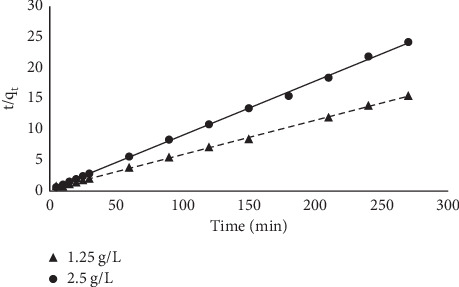
Pseudo-second-order plot for the sorption of MB onto MNS.

**Figure 16:**
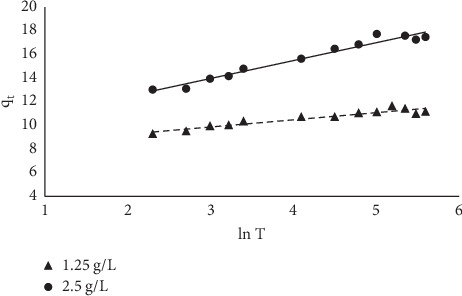
Elovich plot for the sorption of MB onto MNS.

**Figure 17:**
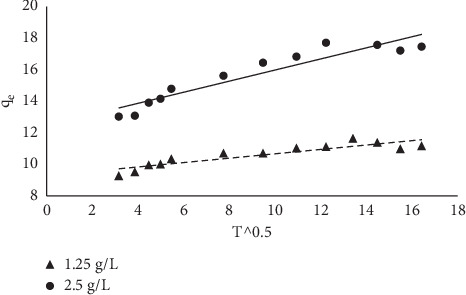
Intraparticle diffusion plot for the sorption of MB onto MNS.

The kinetics of the sorption was studied using four models. The linearized forms of the models were plotted, and the results are presented in Figures 14–17. The sorption can be described by most of the models used because their linear regression line was greater than 0.7; the pseudo-second-order kinetic model best described the kinetics of the sorption. Results obtained from the pseudo-second-order plot suggest that sorption proceeds by chemisorption which is the formation of bonds between the sorbate and the sorbent. This was further supported from the equilibrium model as the Langmuir model assumes a chemisorption process rather than a physisorption process. The kinetic constants obtained from the plots are presented in Table 5.

The intraparticle model by Weber and Morris is more of a mechanistic-based model which is used to ascertain the mechanism of the rate-determining step. If a linear plot of qt versus t0.5 passes through the origin, it implies that the intraparticle diffusion is the sole rate-determining step and if not as obtained in this study, it suggests that the mechanism of the sorption is multilinear and the rate-determining step is controlled by both film diffusion and intraparticle diffusion [29].

## Figures and Tables

**Table 1:** FT-IR band assignment of the sorbent.

IR peaks	Bands (cm^−1^)	Band assignment
1	3623	O-H stretching of alcohols
2	3412	O-H stretching of alcohols
3	3218	O-H stretching of alcohols
4	2911	C-H stretching of alkanes
5	2673	C-H stretching of aldehydes
6	2488	N-H stretching of secondary amine
7	2128	C≡C stretching of alkynes
8	1729	C=O stretching of aldehydes
9	1389	C-H bending of aldehydes

**Table 2:** Brunauer–Emmett–Teller characteristics of pulverized macadamia nut shell.

Physical parameters	Results obtained
BET surface area (m^2^/g)	2.763
Micropore surface area (m^2^/g)	2.938
Total pore volume (cm^3^/g)	0.0108
Micropore volume (cm^3^/g)	0.0105
Average pore diameter (nm)	15.625

**Table 3:** Equilibrium model plot constant for the sorption of MB onto MNS.

Equilibrium models	Temperature		
	303 K	313 K	323 K
Langmuir			
q_max_ (mgg^−1^)	25.77	26.81	27.86
B (l mg^−1^)	0.28	0.33	0.43
*R*^2^	0.93	0.91	0.84
*R*_L_	0.05	0.04	0.03

Freundlich			
*K*_f_ (mg g^−1^) (mg^−1^)^1/*n*^)	1.14	1.11	1.10
1/*n*	0.197	0.192	0.176
*R*^2^	0.85	0.86	0.82

Temkin			
*K*_T_ (mol g^−1^)	94.34	21.83	7.70
*b*_T_ (mol kJ^−1^)	2.79	3.75	21.83
*R*^2^	0.73	0.87	0.88

**Table 4:** Thermodynamics parameters for the sorption of MB onto MNS.

Adsorbent	ΔH (kJ · mol^−1^)	ΔS (kJ · mol^−1^·K^−1^)	ΔG (kJ · mol^−1^)		
			303 K^−1^	313 K^−1^	323 K^−1^
Pulverized MNS	17.45	0.15	−28.72	−30.10	−31.77

**Table 5:** Kinetic model plot constants.

Kinetic models	Concentration	
	1.25 g/L	2.5 g/L
Pseudo first order		
q_e_ (mg/g)	5.27	2.06
K_1_ (min^−1^)	0.0127	7.13 × 10^−3^
*R*^2^	0.872	0.714

Pseudo second order		
q_e_ (mg/g)	17.92	11.34
K_2_ (g/mg min)	8.66 × 10^−3^	0.03
*R*^2^	0.999	0.999

Elovich		
*α* (mg/g min)	7.64 × 10^2^	9.23 × 10^5^
*β*	0.66	1.65
*R*^2^	0.964	0.905

Intraparticle diffusion		
*k*_p_ (mg/g min^1/2^)	0.352	0.129
C	12.46	9.28
*R*^2^	0.89	0.81

## Data Availability

Majority of the data used in this study are included in the article. Other data can be made available upon request from the corresponding author.

## References

[1] Edokpayi J., Odiyo J., Popoola O., Msagati T. (2016). Determination and distribution of polycyclic aromatic hydrocarbons in rivers, sediments and wastewater effluents in Vhembe District, South Africa. *International Journal of Environmental Research and Public Health*.

[2] Edokpayi J. N., Odiyo J. O., Durowoju O. S. (2017). *Impact of wastewater on surface water quality in developing countries: a case study of South Africa. “Water Quality”*.

[3] Aniyikaiye T., Oluseyi T., Odiyo J., Edokpayi J. (2019). Physico-Chemical analysis of wastewater discharge from selected paint industries in lagos, Nigeria. *International Journal of Environmental Research and Public Health*.

[4] Singh R., Singh T. S., Odiyo J. O., Smith J. A., Edokpayi J. N. (2020). Evaluation of methylene blue sorption onto low-cost bio sorbents: equilibrium, kinetics and thermodynamics. *Journal of Chemistry*.

[5] Bharathi K. S., Ramesh S. T. (2013). Removal of dyes using agricultural waste as low-cost adsorbents: a review. *Applied Water Science*.

[6] Edokpayi J. N., Ndlovu S. S., Odiyo J. O. (2019). Characterization of pulverized Marula seed husk and its potential for the sequestration of methylene blue from aqueous solution. *BMC Chemistry*.

[7] Din M. I., Hussain Z., Mirza M. L., Athar M. M., Madri A., Ahmed S. (2013). Biosorption of toxic Congo red dye from aqueous solution by eco- friendly biosorbent Saccharum bengalense: kinetics and Thermo- dynamics. *Desalin Water Treat*.

[8] Yusuf M. (2018). *Handbook of Textile Effluent Remediation*.

[9] Olakunle M. O., Inyinbor A. A., Dada A. O., Bello O. S. (2017). Combating dye pollution using cocoa pod husks: a sustainable approach. *International Journal of Sustainable Engineering*.

[10] Litefti K., Sonia Freire M., Stitou M., González-Álvare J. (2019). Adsorption of an anionic dye (Congo red) from aqueous solutions by pine bark. *Scientific Reports*.

[11] Adegoke K. A., Bello O. S. (2015). Dye sequestration using agricultural wastes as adsorbents. *Water Resources and Industry*.

[12] Kyzas G., Kostoglou M. (2014). Green adsorbents for wastewaters: a critical review. *Materials*.

[13] Gupta V. K., Suhas N. (2009). Application of low-cost adsorbents for dye removal—a review. *Journal of Environmental Management*.

[14] Department of Agriculture, Forestry and Fishery, Republic of South Africa (2012) (2012). *A Profile of the South African Macadamia Nuts Market Value Chain*.

[15] Tam M. S., Antal M. J. (1999). Preparation of activated carbons from macadamia nut shell and coconut shell by air activation. *Industrial & Engineering Chemistry Research*.

[16] Pakade V. E., Maremeni L. C., Ntuli T. D., Tavengwa N. T. (2016). Application of quaternized activated carbon derived from macadamia nutshells for the removal of hexavalent chromium from aqueous solutions. *South African Journal of Chemistry*.

[17] Junior O. P., Cazetta A. L., Gomes C. L. (2014). Synthesis of ZnCl2-activated carbon from macademia nut endocarp (Macademia intergrifolia) by microwave-assisted pyrolysis: optimization using RSM and methylene blue adsorption. *Journal of Analytical and Applied Pyrolysis*.

[18] Martins A. C., Pezoti O., Cazetta A. L. (2015). Removal of tetracycline by NaOH-activated carbon produced from macadamia nut shells: kinetic and equilibrium studies. *Chemical Engineering Journal*.

[19] Rodrigues L. A., de Sousa Ribeiro L. A., Thim G. P., Ferreira R. R., Alvarez-Mendez M. O., Coutinho A. d. R. (2013). Activated carbon derived from macadamia nut shells: an effective adsorbent for phenol removal. *Journal of Porous Materials*.

[20] Du C., Xue Y., Wu Z., Wu Z. (2017). Microwave-assisted one-step preparation of macadamia nut shell-based activated carbon for efficient adsorption of Reactive Blue. *New Journal of Chemistry*.

[21] Langmuir I. (1916). The constitution and fundamental properties of solids and liquids. Part I. Solids. *Journal of the American Chemical Society*.

[22] Freundlich H. (1907). Über die Adsorption in Lösungen. *Zeitschrift für Physikalische Chemie*.

[23] Temkin M., Pyzhev V. (1940). Kinetics of ammonia synthesis on promoted iron catalysts. *Acta Physiochimica. URSS*.

[24] Hoff V. (1884). Studies in dynamic chemistry. https://www.nobelprize.org/prizes/chemistry/1901/hoff/biographical/.

[25] Lagergren S. K. (1898). About the theory of so-called adsorption of soluble substances. *Sven Vetenskapsakad Handingarl*.

[26] Ho Y. S., McKay G. (1998). Kinetic model for lead (II) sorption on to peat. *Adsorption Science & Technology*.

[27] Aharoni C., Ungarish M. (1976). Kinetics of activated chemisorptions. Part I: the non-elovichian part of the isotherm. *Journal of Chemical Society, Faraday Transaction*.

[28] Weber W. J., Morris J. C. (1963). Kinetics of adsorption on carbon from solution. *Journal of the Sanitary Engineering Division*.

[29] Edokpayi J., Odiyo J., Msagati T., Popoola E. (2015). A novel approach for the removal of lead (II) ion from wastewater using mucilaginous leaves of diceriocaryum eriocarpum plant. *Sustainability*.

[30] Zvinowanda C. M., Okonkwo J. O., Sekhula M. M., Agyei N. M., Sadiku R. (2009). Application of maize tassel for the removal of Pb, Se, Sr, U and V from borehole water contaminated with mine wastewater in the presence of alkaline metals. *Journal of Hazardous Materials*.

[31] Lehmann J., Joseph S. (2009). *Biochar for Environmental Management: Science & Technology*.

[32] Lingamdinne L. P., Roh H., Choi Y.-L., Koduru J. R., Yang J.-K., Chang Y.-Y. (2015). Influencing factors on sorption of TNT and RDX using rice husk biochar. *Journal of Industrial and Engineering Chemistry*.

[33] Wekoye J. N., Wanyonyi W. C., Wangila P. T., Tonui M. K. (2020). Kinetic and equilibrium studies of Congo red dye adsorption on cabbage waste powder. *Environmental Chemistry and Ecotoxicology*.

[34] Taher T., Rohendi D., Mohadi R., Lesbani A. (2019). Congo red dye removal from aqueous solution by acid-activated bentonite from sarolangun: kinetic, equilibrium, and thermodynamic studies. *Arab Journal of Basic and Applied Sciences*.

[35] Odiyo J. O., Edokpayi J. N. Physico-chemical and surface characterisation of a renewable low-cost biosorbent for the uptake of heavy metal ions from aqueous solution.

